# Updated Framework for Development of Evidence-Based Recommendations by the Advisory Committee on Immunization Practices

**DOI:** 10.15585/mmwr.mm6745a4

**Published:** 2018-11-16

**Authors:** Grace Lee, Wendy Carr, ACIP Evidence-Based Recommendations Work Group, ACIP Evidence Based Recommendations Work Group, Art Reingold, Paul Hunter, Grace Lee, Jon Temte, Doug Campos-Outcalt, Lorry Rubin, Sean O’Leary, Margot Savoy, Amir Qaseem, Nadine Sicard, Shainoor Ismail, Roshan Ramanathan, Rebecca Morgan, David Weber, Valéry Lavergne, Ned Calonge, Charles Wiysonge, Philippe DuClos, Melanie Marti, Thomas Harder, Gerhard Falkenhorst, Almea Matanock, Tamara Pilishvili, Kathleen Dooling, Carolyn Bridges, Megan Lindley, Tom Shimabukuro

**Affiliations:** ^1^Lucile Packard Children’s Hospital and Professor of Pediatrics, Stanford University School of Medicine, Stanford, California; ^2^Office of the Director, National Center for Immunization and Respiratory Diseases, CDC.; Advisory Committee on Immunization Practices; Advisory Committee on Immunization Practices; Advisory Committee on Immunization Practices; Advisory Committee on Immunization Practices; Advisory Committee on Immunization Practices; Advisory Committee on Immunization Practices; American Academy of Pediatrics; Committee on Infectious Diseases; American Academy of Family Physicians; American College of Physicians; National Advisory Committee on Immunization (Canada; National Advisory Committee on Immunization (Canada; Food and Drug Administration; U.S. GRADE Network; Department of Clinical Epidemiology & Biostatistics, McMaster University; University of North Carolina Schools of Medicine and Public Health; Clinical Practice Guideline; Infectious Diseases Society of America; U.S. Preventive Services Task Force; World Health Organization Strategic Advisory Group of Experts on Immunization; World Health Organization Strategic Advisory Group of Experts on Immunization; World Health Organization Strategic Advisory Group of Experts on Immunization; Standing Vaccination Committee Secretariat, Robert Koch Institute; Standing Vaccination Committee Secretariat, Robert Koch Institute; CDC; CDC; CDC; CDC; CDC; CDC.

The Advisory Committee on Immunization Practices (ACIP)* is a federal advisory committee that provides expert advice to the Director of CDC and the Secretary of the U.S. Department of Health and Human Services in the form of recommendations on the use of vaccines and related agents for control of vaccine-preventable diseases in the U.S. civilian population (*1*,*2*). Work groups that gather, analyze, and prepare scientific information assist in the recommendation formulation process and present options for recommendations based on the scientific evidence they have assessed. Recommendations that are approved by a majority of ACIP's voting members are then reviewed by the Director of CDC and published in *MMWR* if approved by the director. This report briefly summarizes an update to the ACIP process for developing evidence-based recommendations that ACIP adopted at its February 2018 meeting.

In 2010, ACIP formally adopted the Grading of Recommendations Assessment, Development and Evaluation (GRADE) approach for developing evidence-based recommendations (*3*–*6*). Since then, the preparation and presentation of GRADE evidence profiles, which communicate an assessment of the quality of the evidence for outcomes related to benefits and harms (*7*), has been an integral part of ACIP recommendation development (https://www.cdc.gov/vaccines/acip/recs/grade/table-refs.html). However, during the process of recommendation formulation, in addition to the certainty in the evidence as presented in such evidence profiles, panels consider myriad factors such as the values held by the target population regarding the outcomes, health economic data, and implementation issues. Significant additions in this area have been incorporated into GRADE methodology in the past 8 years, particularly the use of Evidence to Decision or Evidence to Recommendation (EtR) frameworks to support the process of moving from evidence to decision and to provide transparency regarding the impact of additional factors on deliberations when considering a recommendation (*8*). Elucidation of these factors and the judgments behind them facilitate transparency, consistency, and communication of recommendations to health care providers, partner organizations, and the public.

ACIP has continued to follow the methodological advances in the GRADE approach, and, as a result, has developed a modified EtR framework tailored to the needs of ACIP (https://www.cdc.gov/vaccines/acip/recs/grade/downloads/ACIP-evidence-rec-frame-508.pdf), which was formally adopted by a unanimous vote at the February 2018 ACIP meeting. Other guideline development panels, including National Immunization Technical Advisory Groups and the World Health Organization’s Strategic Advisory Group of Experts on Immunization, have adopted the new GRADE approach and developed EtR frameworks for use in formulating recommendations (*9*). The ACIP Evidence-Based Recommendations Work Group, which includes internal stakeholders, current and former ACIP members, external methodologists, and representatives from the GRADE Working Group,^†^ is actively engaged in the development and review of the ACIP EtR framework and supporting materials.

New or substantially revised ACIP recommendations for vaccination will use the EtR framework to communicate the deliberations and judgments made by ACIP during formulation of its recommendations. Recommendations will be communicated in the framework in one of three categories: 1) ACIP recommends vaccination for all persons in an age group or a group at increased risk for vaccine-preventable disease; 2) ACIP does not recommend the use of a vaccine; or 3) the ACIP recommendation relies upon guidance of the clinician in the context of individual clinician-patient interactions to determine whether or not vaccination is appropriate for a specific patient. In some instances (e.g., when additional information is needed), ACIP might not make a recommendation, and this option is also reflected in the EtR framework separately (https://www.cdc.gov/vaccines/acip/recs/grade/downloads/ACIP-evidence-rec-frame-508.pdf).

This standardized and more explicit process for developing ACIP recommendations is expected to enhance transparency, consistency, and communication. Additional information about GRADE is available at https://www.cdc.gov/vaccines/acip/recs/grade/about-grade.html.

**Summary**

**What is already known about this topic?**

In 2010, the Advisory Committee on Immunization Practices (ACIP) implemented the Grading of Recommendations Assessment, Development and Evaluation (GRADE) approach for developing evidence-based recommendations.

**What is added by this report?**

Since the original adoption of the GRADE evidence-based recommendation process by ACIP, the use of Evidence to Decision or Evidence to Recommendation (EtR) frameworks have been incorporated into GRADE methodology. ACIP adopted the use of an EtR framework at its February 2018 meeting.

**What are the implications for public health practice?**

The EtR framework elucidates the additional factors considered in vaccine recommendation deliberations and facilitates transparency, consistency, and communication of recommendations to health care providers, partner organizations, and the public.

## Footnotes

* Recommendations for routine use of vaccines in children, adolescents, and adults are developed by the Advisory Committee on Immunization Practices (ACIP). ACIP is chartered as a federal advisory committee to provide expert external advice and guidance to the Director of CDC on use of vaccines and related agents for the control of vaccine-preventable diseases in the civilian population of the United States. Recommendations for routine use of vaccines in children and adolescents are harmonized to the greatest extent possible with recommendations made by the American Academy of Pediatrics (AAP), the American Academy of Family Physicians (AAFP), and the American College of Obstetricians and Gynecologists (ACOG). Recommendations for routine use of vaccines in adults are harmonized with recommendations of AAFP, ACOG, the American College of Physicians (ACP), and the American College of Nurse-Midwives. ACIP recommendations adopted by the CDC Director become agency guidelines on the date published in the *Morbidity and Mortality Weekly Report* (*MMWR)*. Additional information regarding ACIP is available at https://www.cdc.gov/vaccines/acip.

† http://www.gradeworkinggroup.org/.
